# *Brugia malayi* Gene Expression in Response to the Targeting of the *Wolbachia* Endosymbiont by Tetracycline Treatment

**DOI:** 10.1371/journal.pntd.0000525

**Published:** 2009-10-06

**Authors:** Elodie Ghedin, Tiruneh Hailemariam, Jay V. DePasse, Xu Zhang, Yelena Oksov, Thomas R. Unnasch, Sara Lustigman

**Affiliations:** 1 University of Pittsburgh School of Medicine, Pittsburgh, Pennsylvania, United States of America; 2 Lindsley F. Kimball Research Institute, New York Blood Center, New York, New York, United States of America; 3 Department of Global Health, University of South Florida, Tampa, Florida, United States of America; University of Queensland, Australia

## Abstract

**Background:**

*Brugia malayi*, like most human filarial parasite species, harbors an endosymbiotic bacterium of the genus *Wolbachia*. Elimination of the endosymbiont leads to sterilization of the adult female. Previous biochemical and genetic studies have established that communication with its endobacterium is essential for survival of the worm.

**Methodology/Principal findings:**

We used electron microscopy to examine the effects of antibiotic treatment on *Wolbachia* cell structure. We have also used microarray and quantitative RT-PCR analyses to examine the regulation of the *B. malayi* transcripts altered in response to the anti-*Wolbachia* treatment. Microscopy of worms taken from animals treated with tetracycline for 14 and 21 days (14 d and 21 d) demonstrated substantial morphologic effects on the *Wolbachia* endobacterium by 14 d and complete degeneration of the endobacterial structures by 21 d. We observed upregulation of transcripts primarily encoding proteins involved in amino acid synthesis and protein translation, and downregulation of transcripts involved in cuticle biosynthesis after both 7 d and 14 d of treatment. In worms exposed to tetracycline in culture, substantial effects on endobacteria morphology were evident by day 3, and extensive death of the endobacteria was observed by day 5. In a detailed examination of the expression kinetics of selected signaling genes carried out on such cultured worms, a bimodal pattern of regulation was observed. The selected genes were upregulated during the early phase of antibiotic treatment and quickly downregulated in the following days. These same genes were upregulated once more at 6 days post-treatment.

**Conclusions/Significance:**

Upregulation of protein translation and amino acid synthesis may indicate a generalized stress response induced in *B. malayi* due to a shortage of essential nutrients/factors that are otherwise supplied by *Wolbachia*. Downregulation of transcripts involved in cuticle biosynthesis perhaps reflects a disruption in the normal embryogenic program. This is confirmed by the expression pattern of transcripts that may be representative of the worms' response to *Wolbachia* in different tissues; the early peak potentially reflects the effect of bacteria death on the embryogenic program while the second peak may be a manifestation of the adult worm response to the affected bacteria within the hypodermis.

## Author Summary

Filarial parasites afflict hundreds of millions of individuals worldwide, and cause significant public health problems in many of the poorest countries in the world. Most of the human filarial parasite species, including *Brugia malayi*, harbor endosymbiotic bacteria of the genus *Wolbachia*. Elimination of the endosymbiont leads to sterilization of the adult female worm. The need exists for the development of new chemotherapeutic approaches that can practically exploit the vulnerability of the filaria to the loss of the *Wolbachia*. In this study we performed ultrastructural and microarray analyses of female worms collected from infected jirds treated with tetracycline. Results suggest that the endosymbiotic bacteria were specifically affected by the antibiotic. Furthermore, in response to the targeting of the endosymbiont, the parasites modulated expression of their genes. When exposed to tetracycline, the parasites over-expressed genes involved in protein synthesis. Expression of genes involved in cuticle biosynthesis and energy metabolism was, on the other hand, limited.

## Introduction

Nematodes are the most common parasitic infections of humans [1] with filarial worms infecting more than 150 million individuals worldwide [2],[3]. *Brugia malayi* and *Wuchereria bancrofti* are filarial parasites of the lymphatic and circulatory system, and infection with these parasites can result in lymphatic pathologies leading to elephantiasis. In contrast, *Onchocerca volvulus* resides in the connective tissue and dermis, and infection can result in onchocerciasis, or river blindness and severe skin disease, or onchodermatitis. Approximately 30 years ago, bacteria-like structures residing within cells of the filarial parasites were first observed [4],[5]. Experiments suggested that treatment with certain antibiotics that target bacteria, including tetracycline and chloramphenicol, could interrupt the third-stage (L3) to fourth-stage (L4) larval molt of the worm [6] and also affect embryogenesis in adult parasites [7],[8]. Later studies revealed the presence of *Wolbachia* DNA in the majority of filaria, including all of the human parasites with the exception of *Loa loa* [9],[10] and *Mansonella perstans* [9]. Treatment with both ivermectin (the microfilaricidal drug that is the major tool for onchocerciasis control today) and the antibiotic doxycycline resulted in a rapid and apparently irreversible decline in the burden of skin microfilariae in *O. volvulus*-infected individuals and an apparent permanent sterilization of the adult female parasite [11]. Studies on *W. bancrofti* infections suggest that a doxycycline treatment regimen can successfully target worm embryogenesis and stop microfilarial production in individuals infected with this parasite as well [12]. Although the doxycycline treatment studies demonstrate an effect on *Wolbachia* – and consequently on the survival of the filaria – the use of this antibiotic is not a practical choice for the treatment of human filarial infections. First, doxycycline is a relatively toxic drug that is contraindicated in a fairly large number of patient groups, including children and pregnant or lactating women. Second, clearance of the *Wolbachia* endosymbiont requires a very prolonged antibiotic treatment course, something that is impractical in developing countries where human filarial infections are endemic. Finally, studies employing other antibiotics generally used to treat endocellular bacterial infections were shown to be uniformly ineffective against filarial *Wolbachia* [13]. For these reasons, additional research is critically needed to develop new tools for control and treatment of these infections. If the *Wolbachia* endosymbiont is to be exploited as a practical chemotherapeutic target, a better understanding of its effects on its host is crucial.

In the present study we focused on the bacteria-host relationship by characterizing the effect of eliminating the *Wolbachia* of *B. malayi* (w*Bm*) on worm gene expression. Transcriptomic approaches have proven useful in studying gene expression patterns in other parasites [14],[15], as well as the host response to pathogens [16]. Previous studies have shown that tetracycline has no direct effect on filarial parasites that do not carry an endosymbiont, such as *Acanthocheilonema viteae* [8]. However, similar to what was seen when doxycycline was given to *W. bancrofti* and *O. volvulus*-infected humans, tetracycline treatment of animals infected with *Litomosoides sigmodontis* (an endosymbiont-containing filarial parasite commonly used as a model for the human filaria) resulted in the elimination of the endosymbiont and reduced the fertility of the adult female worm [8]. While prolonged treatment with tetracycline is effective for the elimination of the endobacteria, the specific molecular effects of the affected bacteria on fertility and molting in its parasite host are unknown. We hypothesized that *B. malayi* genes that respond early to w*Bm* clearance are likely to have an important role in the symbiotic relationship. As a first step in understanding this process we set out to identify genes whose expression is altered early in response to the targeting of the w*Bm* endosymbiont by tetracycline treatment.

## Materials and Methods

### Drug Treatment of *B. malayi* worms

#### Ethics statement

The *in vivo* treatment was carried out under protocol #R3230 approved by the University of South Florida's Institutional Animal Care and Use Committee (IACUC).

#### Drug treatment

Adult *B. malayi* were subjected to antibiotic treatment to eliminate their endosymbionts using both *in vivo* and *in vitro* approaches. In the *in vivo* treatment experiments, jirds (*Meriones unguiculatus*) infected 120 days earlier with *B. malayi* were obtained from the NIAID/NIH Filariasis Research Reagent Repository Center (FR3; Athens, GA; www.filariasiscenter.org) and were treated with 2.5 mg/ml tetracycline in drinking water for 7, 14 or 21 days as previously described [17]. Age-matched control female worms were recovered from infected jirds given normal water. Necropsies and worm recoveries were performed at the end of each treatment period. Worms were sexed, washed and 8–10 female worms per group were immediately processed for RNA or fixed for electron microscopic analysis, as described below.

*In vitro* treatments were carried out on parasites maintained for short periods in culture. To accomplish this, female worms were obtained from animals (FR3) at 120 days post-infection. The isolated worms were washed several times with RPMI-1640 (GIBCO, Grand Island, New York) containing 100 U/ml streptomycin, 100 µg/ml penicillin, and 0.25 mg/ml of amphotericin-B (Sigma), and then cultured in groups of 4 in 2 ml of complete media (CM; RPMI 1640 supplemented with 25 mM HEPES buffer, 2 mM glutamine, 100 U/ml streptomycin, 100 µg/ml penicillin, 0.25 µg/ml of amphotericin B, and 10% heat-inactivated fetal calf serum) or with 40 µg/ml tetracycline (Sigma) in CM in 24-well culture plates (Costar, Cambridge, Massachusetts). In a previous study, treatment *in vitro* with 40 µg/ml tetracycline was shown to be the minimum effective concentration capable of reducing microfilarial release by ∼100% by day 10 [18]. Cultured female worms were incubated at 37°C in an atmosphere of 5% CO_2_ in air. The culture medium was replaced daily with CM or CM with tetracycline. All cultures were terminated on day 7. The macroscopic effect of the antibiotic treatment on microfilarial release and viability was evaluated by viewing the treated parasites with an inverted microscope. Motility was scored at each time point by comparing to female worms cultured in CM in the absence of antibiotics. The worm cultures with or without antibiotics at each time point were carried out in triplicate or quadruplicate. One set was used for electron microscopic studies and the other two or three sets for quantitative reverse transcriptase PCR (qRT-PCR).

### Microarray hybridization and expression analyses using RNA isolated from *B. malayi* worms

#### Microarray

We used the Filarial Nematode Oligonucleotide Array-Version 2 (BmV2array) [19] which contains unique 55-mer oligonucleotides corresponding to 15,455 *B. malayi* ORFs and ESTs as well as 1,016 *O. volvulus*, 878 *W. bancrofti* and 804 *Wolbachia* (w*Bm*) annotated or predicted genes (www.filariasiscenter.org). RNA was extracted using TRIzol reagent (Invitrogen, Carlsbad, CA) from 8–10 worms treated with tetracycline for 7 or 14 days and from age-matched control worms. Total RNA was prepared according to the manufacturer's instructions. Total RNA quality was determined by an Agilent 2100 bioanalyzer (Agilent Technologies, Palo Alto, CA) according to manufacturer's recommendations.

Hybridization and analyses were done at the Genome Sequencing Center of the Washington University School of Medicine, St. Louis. First strand cDNA was generated by oligo-dT primed reverse transcription (Superscript II; Invitrogen) utilizing the 3DNA Array 900 kit (Genisphere). One µl of Cy3 or Cy5 fluorophore-labeled oligo-dT primer was added to 2 µg of total RNA in a total volume of 5.5 µl and the solution was incubated at 80°C for 5 minutes and cooled on ice for 2 minutes. To each sample, 0.5 µl of RNase inhibitor (Superase-In; Ambion) (0.5 µl), 2 µl of 5× first strand buffer, 0.5 µl of dNTP mix (10 mM each dATP, dCTP, dGTP, and dTTP), 1 µl of 0.1 M DTT and 0.5 µl of Superscript II RNase H- Reverse Transcriptase were added. Reverse transcription was carried out at 42°C for 2 hours. The reaction was terminated by adding 1 µl of 1.0 M NaOH/100 mM EDTA followed by incubation at 65°C for 10 minutes. The samples were then neutralized by the addition of 1.2 µl of 2 M Tris-HCL, pH 7.5. The resulting labeled cDNAs were used as hybridization probes against the *B. malayi* microarray BmV2array. Each microarray was hybridized with a mixture of control and experimental cDNA differentially labeled with Cy3 and Cy5. To ensure that any effect noted was not the result of differential labeling by a particular dye, parallel experiments were carried out in which the dyes used to label paired control and experimental RNA preparations were switched (“flip-dye” replicates).

Hybridizations were performed as previously described [20]. Slides were scanned immediately after hybridization. Gridding and analysis of images were performed using ScanArray v3.0 (Perkin Elmer). Each spot was defined on a pixel-by-pixel basis, using a modified Mann-Whitney statistical test. The resulting values were imported into Gene-Spring 7.1 software (Agilent, Redwood city, CA). Local background intensity was subtracted from individual spot intensities. A Lowess curve was fit to the log-intensity versus log-ratio plot. Twenty percent (20.0%) of the data was used to calculate the Lowess fit at each point. This curve was used to adjust the control value for each measurement. If the control channel was lower than 10 RFU a value of 10 was substituted for the observed value. Signal to Lowess adjusted controlled ratios were calculated. Data were filtered using the Student's t-test function in GeneSpring. Genes with differences corresponding to a *P*-value of <0.05 and which also had signal to control or control to signal ratios >2.0 were considered to exhibit a significant up- or downregulation relative to the untreated worms.

Technical replicates were averaged and data was filtered independently for each time point. For each time point to be included in the final analysis, it had to meet three criteria. First, genes had to be “present” in 2 of the 4 hybridizations. Second, genes had to be greater than 500 rfu in either channel (raw) in 2 of the 4 hybridizations. Third, genes had to exhibit a two fold or greater change in either direction in at least one of the two technical flip-dye replicates. Genes were filtered according to a t-test *P* -value between 0 and 0.05 in at least one of the two conditions. The cross-gene error model was inactive. Multiple-testing correction was not applied. The data from the microarray experiments reported here have been submitted to ArrayExpress, accession E-MEXP-2185.

#### qRT-PCR

For RNA extraction, female worms were snap frozen, crushed in liquid nitrogen using a mortal and pestle. The samples were then re-suspended in TRIzol reagent (Invitrogen, Carlsbad, CA) and total RNA was prepared according to the manufacturer's instructions. The quantity of RNA was measured with a GeneQuant Spectrophotometer (Pharmacia Biotech, Piscataway, NJ). An aliquot of total RNA from the same sample used for the 7 d microarray hybridizations as well as additional RNA samples collected from other biological replicates prepared from worms collected from animals exposed to tetracycline for 7 d or 14 d were used for the qRT-PCR studies. Just prior to proceeding with cDNA synthesis, RNA samples were treated with amplification grade DNase I (Gibco BRL, Gaithersburg, MD) to eliminate any genomic DNA contamination.

First-strand complementary DNA was synthesized from 1 µg total RNA using SuperScript™ III First-Strand Synthesis System for RT-PCR and RNase H-Reverse Transcriptase (Invitrogen, Carlsbad, CA) using oligo (dT)12–18 primer (Promega, Madison, WI). For the PCR, 1 µl of template cDNA was mixed with 12.5 µl SYBR Green/Rox PCR master mix (SABiosciences) and 1 µl each of 5 µM forward and reverse gene-specific primers in a total of 25 µl reaction. The primers were designed using Primer3 [21] to amplify 100–150 bp of target transcripts. The list of primers is available in Table S1. The PCR conditions used were: 50°C for 2 min, 95°C for 10 min and 40 cycles of 15 seconds at 95°C, 30 seconds at 60°C and 30 seconds at 72°C on the ABI 7300 Real-time PCR system. To characterize amplification specificity, a melting curve analysis was performed at the end of the 40th cycle. Threshold cycle values were then calibrated against *B. malayi* NADH dehydrogenase subunit 1 (NADH), as previously described [22]. We tested the expression levels of this gene in comparison to two other candidate internal control genes, beta actin and histone 3, and found that it was the most consistent in its expression levels in RNA prepared from worms cultured *in vitro* for 1–7 days with or without tetracycline.

The relative expression of each transcript in the treated worms vs. adult female control worms was calculated using the ΔΔC_t_ method. This method involves comparing the C_t_ values of the samples of interest with a control or calibrator such as a non-treated sample, and where ΔΔC_t_ = ΔC_t (treated)_−ΔC_t (control)_. ΔC_t_ is the C_t_ value for any sample, treated or untreated, normalized to the endogenous housekeeping gene, NADH. Then ΔΔC_t_ values were calculated by subtracting the ΔC_t_ value of the control group from that of tetracycline treated samples. Finally, fold change of transcript level in the tetracycline treated samples over the control worms were expressed using the formula 2^−ΔΔCt^.

To verify that tetracycline treatment had a direct effect on the w*Bm*, we tested for *Wolbachia*-RNA expression by qRT-PCR using primers targeting the *Wolbachia*-specific ankyrin gene (wBM0287), using a variation on a previously described protocol [23] in which first-strand cDNA was synthesized from the total RNA using random hexamers. The subsequent procedure for qRT-PCR was as described above. The w*Bm* ankyrin Ct values were standardized against the *B. malayi* gene NADH dehydrogenase subunit 1. Fold change in the ankyrin transcript levels between control and tetracycline treated worms were calculated using the ΔΔC_t_ method described above.

#### Annotation

A KEGG (Kyoto Encyclopedia of Genes and Genomes [24]) analysis was performed to functionally classify the regulated genes [25]. The KEGG pathways identified for each treatment set are listed in Table S4. To generate Gene Ontology (GO) associations for differentially expressed genes detected in the microarray experiments, we identified enriched GO terms. A list of GO terms relevant for *B. malayi* was then generated using the InterProScan tool [26] and used for comparative analyses of 7 d and 14 d post-treatment datasets. The Gene Ontology Enrichment Analysis Software Toolkit (GOEAST [27]), a web-based software package, was used to discover significantly enriched GO terms in the treatment samples. The *P*-value for the enrichment of each GO term in the microarray dataset was then calculated (Table S5) and the false discovery rate was controlled [28].

### Structural analyses by electron microscopy of treated *B. malayi* worm tissue

For morphological studies, *B. malayi* female worms were fixed with 3% glutaraldehyde in 0.1 M sodium cacodylate buffer, pH 7.4, for 2 hours at room temperature, post-fixed in 1% osmium tetroxide and then grouped within a 3.5% Sea Plaque agar pad. The worms were then dehydrated in graded ethanol solutions (50–100%), embedded in EMbed-812 media, and cured for 24 hours at 56°C. Ultrathin sections (65–70 nm) were cut on an MT-XL ultra-microtome and stained with Uranyl Acetate and Reynold's Lead Citrate. All reagents were from the EMS Company (Hatfield, PA). Samples were observed using a Philips EM-410 Transmission Electron Microscope (Phillips/FEI Corporation, Eindhoven, Holland) at an accelerating voltage of 80 kV.

## Results and Discussion

### *Wolbachia* are cleared from *B. malayi* female worms recovered from 21 day tetracycline-treated jirds

**Figure 1:**
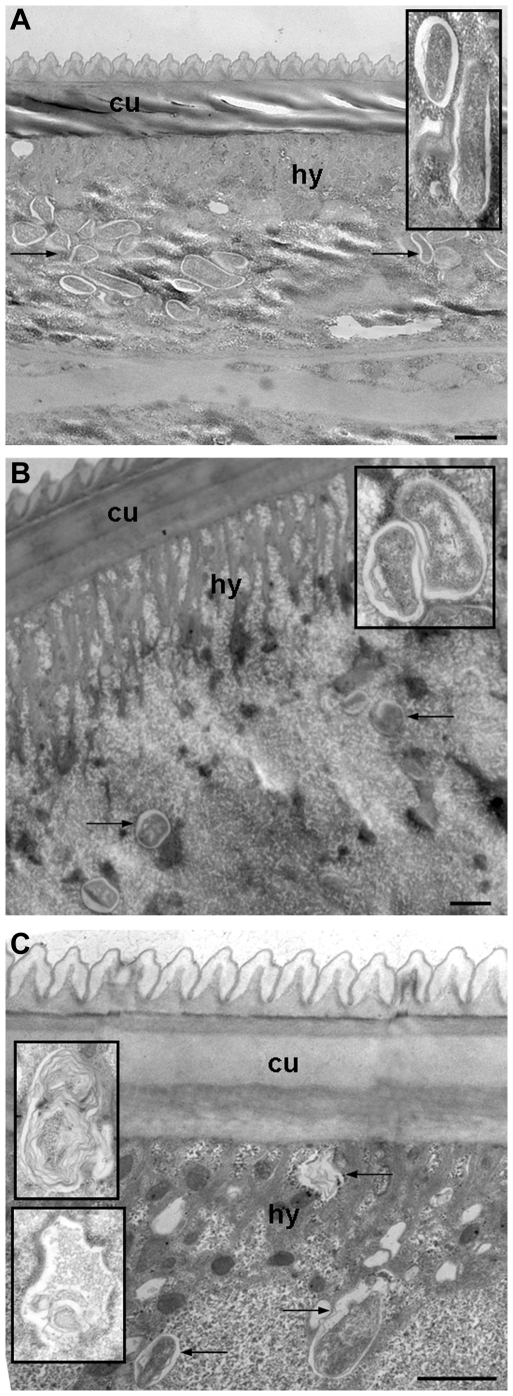
Ultrastructural observation of w*Bm* within the hypodermis of normal and tetracycline treated *B. malayi* adult female worms. Microscopy was carried out as described in Materials and Methods. In comparison to normal worms (A) the number of *Wolbachia* was reduced in worms treated for 14 days (B) or 21 days (C) with 2.5 mg/ml tetracycline (arrows point to *Wolbachia*). In addition, the condition of the surviving *Wolbachia* became worse in the treated worms. In the 14 d treated worms the *Wolbachia* appear to be degenerated (see insert in B), while in 21 d treated worms the endobacteria appear dead and the vacuoles contained only remnants of bacteria or membrane whorls (see inserts in C). The bar is 1000 nm. cu = cuticle; hy = hypodermis.

We conducted an ultrastructural analysis to determine the effect of tetracycline on the endosymbiont after *in vivo* drug treatment. Infected jirds were exposed to 2.5 mg/ml of tetracycline in their drinking water for 14 or 21 days (14 d and 21 d). *B. malayi* worms were collected from treated and control hosts for ultrastructural studies. In female worms from control hosts, large numbers of w*Bm* were present within the hypodermal cord (Figure 1A). In parasites recovered from animals treated for 14 d (Figure 1B) or 21 d (Figure 1C), degenerating *Wolbachia* could be seen in the hypodermis of the worms (see insert in each panel) and the total number of bacteria present appeared to decline compared to the untreated worms. In addition, while the *Wolbachia* were also present in all stages of the uterine progeny of the untreated worms, no w*Bm* were observed in the oocytes, embryos, or in the microfilariae within the worms collected from the treated animals (data not shown). In the 21 d treated worms, many of the bacteria within the hypodermis were also degenerated, and the cell vacuoles contained only remnants of bacteria or membrane whorls (see insert in Figure 1C). A similar observation was made in tetracycline-treated *O. ochengi* where the *Wolbachia* were eliminated resulting in the resolution of the filarial infection [29]. These morphological changes are consistent with ultrastructural images of killed bacteria [30]. Therefore, it was concluded that 21 days of tetracycline treatment of the *B. malayi*-infected jirds would lead to complete w*Bm* cell death. Based on these results, we determined that 14 days of drug exposure was likely to be the maximum time for an efficient evaluation of the effect unhealthy *Wolbachia* have on their host. A set of experiments (ArrayExpress E-MEXP-2185; Source Name 012607) performed using random primed RNA isolated from parasites collected from treated animals (in order to capture bacterial transcripts in addition to nuclear *B. malayi* transcripts), confirmed that at 14 d w*Bm* were dying as the vast majority of their transcripts were found to be downregulated compared with *wBm* from untreated worms. Of the 295 hybridized oligos (*P*-value<0.05) corresponding to endosymbiont encoded genes, 94% indicated transcript downregulation by more than 2-fold, while only 4% were upregulated (Table 1), consistent with the hypothesis that tetracycline treatment results in the death of the *Wolbachia* endosymbiont. In contrast, 36% of *B. malayi* nuclearly-encoded transcripts were downregulated more than 2-fold, while 55% were upregulated more than 2-fold (data not shown). Notably, some of the w*Bm* transcripts that were upregulated at 14 d encoded chaperone proteins and two proteins involved in cytochrome c biogenesis (Table 1).

**Table 1:** Fold increase in the expression levels of *Wolbachia* transcripts found in *Brugia malayi* worms treated for 14 days *in vivo* with tetracycline.

Systematic Oligo_ID	Gene Name	Fold change above control	Annotation
BMX13805	**Wbm0033**	2.05	hypothetical protein
BMX14424	**Wbm0656**	2.17	Methionyl-tRNA formyltransferase
BMX14069	**Wbm0301**	2.19	Uncharacterized protein conserved in bacteria
BMX14402	**Wbm0634**	2.31	ABC-type transport system involved in cytochrome c biogenesis, permease component
BMX14156	**Wbm0390**	2.33	Multisubunit Na+/H+ antiporter, MnhB subunit
BMX13865	**Wbm0094**	2.42	hypothetical protein
BMX13907	**Wbm0137**	2.42	hypothetical protein
BMX14262	**Wbm0495**	2.80	Molecular chaperone, DnaK
BMX14166	**Wbm0400**	3.30	ATP-NAD kinase
BMX14116	**Wbm0349**	3.43	Co-chaperonin GroES (HSP10)
BMX14117	**Wbm0350**	4.43	Chaperonin GroEL (HSP60 family)
BMX14433	**Wbm0665**	4.78	hypothetical protein
BMX14118	**Wbm0351**	6.45	Uncharacterized protein predicted to be involved in C-type cytochrome biogenesis

### *B. malayi* transcripts are differentially expressed in worms treated with tetracycline

In order to capture the effect of tetracycline on gene expression before complete bacteria death and clearance, a second set of microarray experiments was performed. A number of biological replicates were carried out employing groups of parasites collected from animals exposed to tetracycline for 7 d or 14 d. RNA was isolated from groups of 8–10 worms and used in these microarray experiments. In order to focus specifically on *B. malayi* gene expression, the experiments were conducted using oligodT-primed RNA. In analyzing the results, we concentrated upon one set of microarray experiments (ArrayExpress E-MEXP-2185; Source Name 011508 - referred herein as 011508) in which the RNA used was collected at both time points (7 d and 14 d) during the course of the same treatment experiment. In other experiments, worms were collected from groups of animals treated for either 7 d or 14 d but not at both time points. These experiments were, however, used to provide biological replicate support to the conclusions drawn from the 011508 experimental data.

The initial analysis of the data collected from experiment 011508 indicated that expression of only a relatively limited number of *B. malayi* genes was affected by exposure to tetracycline. At 7 d post-treatment in the 011508 data, 212 oligos on the array indicated upregulation of their corresponding transcripts while 51 oligos indicated downregulated transcripts (Table S2A). At 14 d post-treatment, 285 oligos on the array indicated upregulation of their corresponding transcripts, while 34 indicated downregulation (Table S2B). At 7 d, upregulation was predominantly restricted to *B. malayi* genes involved in translation, such as ribosomal proteins (40S ribosomal proteins S4 and S23 and 60S ribosomal proteins L3, L4, L5, L10, L14, L22, L24), the eukaryotic translation initiation factor eIF5A-2 (15549.m00017/Bm1_57565), and the alpha (14894.m00090/Bm1_27170) and gamma (14992.m11155/Bm1_51865) subunits of the translation elongation factor 1 (EF-1).

A subset of genes appeared to be substantially upregulated in response to tetracycline. These were characterized by treated/untreated ratios of greater than 3.0 in the microarray data. These strongly regulated genes included the *B. malayi* peptidyl-prolyl cis-trans isomerase (15451.m00017/Bm1_56870) which is important for cuticular collagen processing [31], and a homologue of a highly immunogenic Gln-rich protein of *O. volvulus* (gb#:AAC48290.1). However, most of the upregulated transcripts encoded proteins involved in regulated degradation of intracellular proteins. These included a member of the serpin family, Bm-SPN-2 (gb#:AAB65744.1), peptidases such as the cathepsin L-like cysteine protease *Bm-cpl-3* (12633.m00021/Bm1_02075), the hydrolase alpha amylase (12556.m00067/Bm1_01300), a ubiquitin-conjugating enzyme family protein (14990.m08098/Bm1_49860), a 26S proteasome non-ATPase regulatory subunit (14971.m02852/Bm1_36060), and asparaginyl-tRNA synthetase (14971.m02889/Bm1_36235). Together, these data point towards *B. malayi* going into increased synthesis of amino acids and protein translation in response to the death of its endosymbiont. Such upregulation of proteins involved in protein synthesis (such as the ribosomal proteins) has been described in several other organisms placed under stress, including bacteria [32], plants [33] and mammalian cells [34]. The upregulation of these mRNAs may indicate a generalized stress response induced in *B. malayi* due to shortage of essential nutrients/factors that are otherwise supplied by w*Bm*. Downregulated genes at 7 d post-treatment included the *B. malayi* superoxide dismutase (gb#AAR06638.1) and cuticular collagens such as an alpha-1 collagen type IX (15377.m00007/Bm1_56350), the nematode cuticular collagen (15378.m00020/Bm1_56360), and other putative collagens (12495.m00012/Bm1_00775, 14845.m00009/Bm1_26670). These genes are involved in cuticle biosynthesis and perhaps reflect a disruption in the normal embryogenic program. The majority of the genes involved in energy metabolism also appeared to be downregulated at 7 d post-treatment. These included transcripts encoding proteins located in the inner mitochondrial membrane and involved in the respiratory chain, such as cytochrome c oxidase subunit II (gb#AAN17813.1), ATP synthase F0 subunit 6 (gb# AAN17812.1), and NADH dehydrogenase subunit 4L (gb# AAN17810.1). Interestingly, in contrast to this general pattern, cytochrome c oxidase subunit IV (12902.m00232/Bm1_03920) was upregulated above the three-fold level. This is of interest as tetracycline was shown to affect host mitochondrial metabolism and reduce cytochrome c oxidase in insects that carry *Wolbachia* [35]. This observation may reflect some direct effect of the antibiotic on host metabolism rather than an indirect effect due to bacteria death.

**Figure 2:**
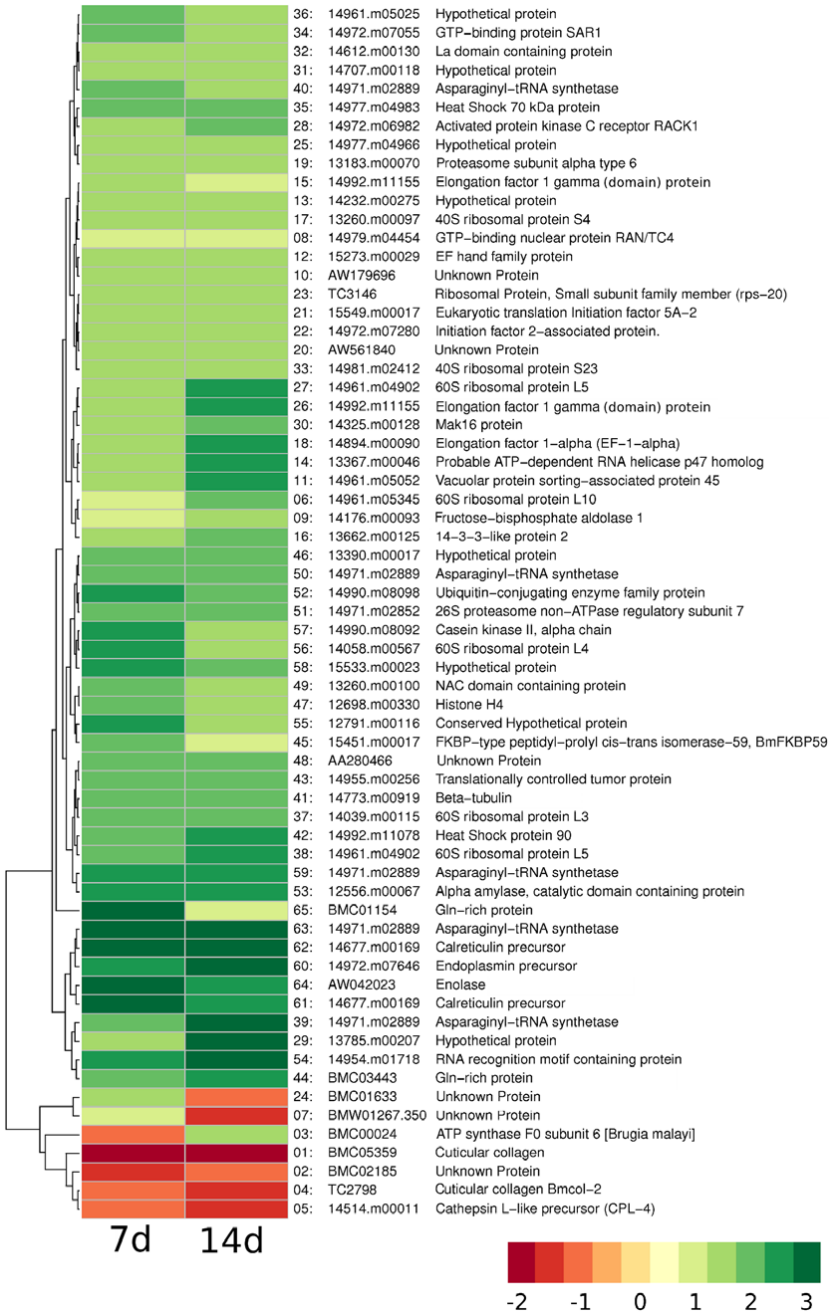
Microarray expression of genes in worms collected from animals given tetracycline for 7 d and 14 d. Only oligos that had an expression value across both time points are represented. Heat map representation of expression levels reported as log_2_ ratios rather than as fold expression levels. Upregulation is shown in green and downregulation in red. Genes are sorted by clustering of gene expression levels.

Figure 2 provides an overview of genes whose expression was found to be changed at both 7 d and 14 d as a result of exposure to tetracycline. At 14 d post-treatment, various transcripts corresponding to proteins involved in translation and in amino acid synthesis (for example ribosomal proteins, elongation factors, and asparaginyl-tRNA synthetase) continued to be overexpressed (Table S2B). Similarly, the majority of downregulated genes seen at 14 d were those involved in cuticle biosynthesis, including the cuticular collagens and cuticular glutathione peroxidase. The transcript encoding asparaginyl-tRNA synthetase was also constantly upregulated at both 7 d and 14 d (Figure 2; details in Table S3). This enzyme was shown in *B. malayi* to be immunodominant and to activate a strong human immunoglobulin G3 response that is thought to contribute to the chronic inflammation seen in lymphatic filariasis [36]. Upregulation of such an immunogenic protein might contribute to the clearance of microfilaria in tetracycline treated individuals. Alternatively, the increased production of such immunogens might temporarily exacerbate the pathologies associated with these infections, since much of the pathology is believed to be immune mediated [37].

Contrary to what was observed at 7 d, at 14 d the majority of the genes that were strongly over-expressed (characterized by treated/untreated ratios of greater than 3.0) corresponded to various proteins located in the inner mitochondrial membrane and involved in the respiratory chain. These include cytochrome c oxidase subunits I (gb#AAN17806.1) and III (gb#AAN17809.1), NADH dehydrogenase subunits 2 (gb#AAN17804.1), and 5 (gb#AAN17815.1), and cytochrome b (gb#AAN17808.1). ATP synthase F0 subunit 6 (gb#AAN17812.1) was also upregulated but at a lower level.

Other genes that are seen to be over-expressed at 14 d and not at 7 d included a trypsin family protein (14979.m04643/Bm1_45620), a superoxide dismutase (gb#AAR06638.1), an ecdysone receptor homolog (14944.m00537/Bm1_29350), and a cathepsin L-like non-peptidase homolog (#BAD11761.1). *Bm-cpl-6*, a member of the group 1c of cathepsin L-like cysteine proteases, was downregulated at 7 d, but upregulated at 14 d. Interestingly, another member of this family, *Bm-cpl-4*, (which belongs to group 1a), was downregulated at both 7 d and 14 d post-treatment. The proteins that belong to group 1a of the cathepsin-L like proteases have been studied extensively because of their function during embryogenesis and larval development in filarial parasites [38]–[40].

Another group of genes of interest included those which were significantly upregulated at 7 d and then downregulated at 14 d. These could be characterized as ‘early responders’ and are most likely to be involved in a direct effect of *Wolbachia* disruption on *B. malayi* transcriptome. This is the case for the excretory/secretory protein Juv-p120 precursor that is similar to the 120 kDa antigen produced by the juvenile female of *Litomosoides sigmodontis* [41]. At 7 d, oligos corresponding to two full length genes are upregulated (14597.m00048/Bm1_21750 and 14478.m00108/Bm1_19955) (Table S2A) while at 14 d an oligo corresponding to a truncated version of the protein is downregulated (12501.m00019/Bm1_00865) (Table S2B). The physiological role of this protein family is uncertain.

**Figure 3:**
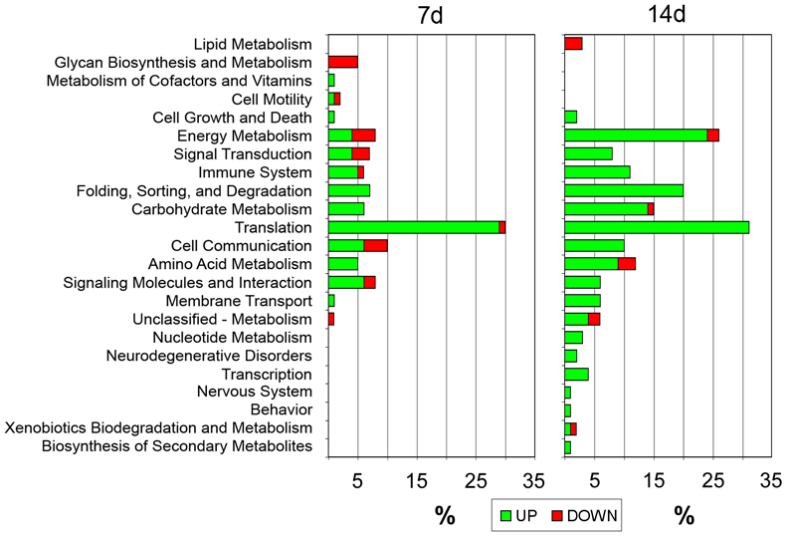
KEGG classification of *B. malayi* proteins regulated in response to tetracycline treatment, 7 days (7 d) and 14 days (14 d) post-treatment. Values for each category are reported as percentages of functionally annotated proteins. Upregulation is shown in green and downregulation in red.

The KEGG classification helps to highlight the most important *B. malayi* pathways that are affected by the tetracycline treatment. From the KEGG analysis (Figure 3; Table S4) it appears that of the upregulated pathways, translation accounts for 30% of functionally annotated proteins both at 7 d and 14 d post-treatment. Energy metabolism is also highly upregulated at 14 d (25% of functionally annotated genes), primarily oxidative phosphorylation (cytochrome c oxidase and NADH dehydrogenase genes) likely due to an effect of w*Bm* death on mitochondria and potentially leading to cytochrome c-mediated apoptosis [42].

To confirm the upregulation or downregulation trends observed in the microarray experiments, qRT-PCR assays for specific genes were performed. In conducting these studies the same RNA preparation used in the 7 d microarray experiment was tested, as well as RNA from biological replicates consisting of worms recovered from other animals at 7 d or 14 d post-treatment. In choosing the genes to be included in these confirmatory assays, we used the KEGG annotation to select genes that corresponded to signaling molecules and the signal transduction pathway. We hypothesized that the regulation of this pathway was likely to reflect an early response to *Wolbachia* cell death.

Results of the qRT-PCR agreed well with those obtained from the microarray experiments (Table 2). In the worms collected from animals given tetracycline for 7 d, the qRT-PCR and microarray data agreed for 14/15 (93%) of the genes tested. The only exception was BMC05356, which appeared to be downregulated in the microarray analysis while slightly upregulated in the qRT-PCR assay. For the RNA samples collected from parasites in animals given tetracycline for 14 d, the microarray and qRT-PCR data agreed for 12/15 (75%) of the genes tested. Again, the microarray data indicated that BMC05356 was downregulated at 14 d, while the qRT-PCR indicated upregulation of this transcript. Similarly, the microarray data suggested that expression of Bm1_15985 and Bm1_26670 were not changed by tetracycline treatment at 14 d, while the qRT-PCR suggested that both genes were in fact upregulated.

**Table 2:** qPCR experiments to confirm expression of signaling genes compared to microarray expression levels. Values in bold correspond to genes for which the qPCR expression data did not support the microarray data.

Locus	annotation	annotation id	Microarray 7 d^a^ *	qPCR 7d-1^a^ **	qPCR 7d-2^b^	Microarray 14 d^c^	qPCR 14d-1^c^	qPCR 14d-2^c^
Bm1_14145	protein phosphatase PP2A regulatory subunit	BMC11777	4.4^d^	33.8	68.6	5.1	13.5	39.7
Bm1_18515	ADP,ATP carrier protein, heart/skeletal muscle isoform T1	BMC11895	1.5^d^	3.5	2.9	3.9	1.8	4.3
Bm1_42375	heavy neurofilament protein	BMC00257	8.0	3.4	2.1	1.5^d^	6.9	9.6
Bm1_43675	heat shock 70 kDa protein	BMC00470	3.1	7.2	5.7	3.3	3.9	7.1
Bm1_48905	Inositol-1	AA480718	1.8^d^	1.9	1.3	3.06	1.4	2.7
Bm1_49830	Casein kinase II, alpha chain	BMC00914, WB-contig_1039	4.8	17.8	29.2	2.9	21.9	26.0
**BMC05356**	**gb|AAA81700.1| Hypothetical protein F13D11.3 [Caenorhabditis elegans]**	**BMC05356**	**0.4**	**4.5**	**6.1**	**0.5** d	**5.8**	**8.9**
BMC00071	cytochrome c oxidase subunit II	BMC00071	0.5	0.9	0.8	0.8^d^	0.8	0.8
Bm1_08420	kinesin light chain protein 2, isoform a	BMC03573	4.1^d^	37.5	353.0	2.8	112.7	159.5
**Bm1_15985**	**hypothetical protein**	**14228.m00020**	3.8	44.1	33.8	**0.9** d	**7.5**	**32.3**
**Bm1_26670**	**collagen**	**14845.m00009**	0.3	1.1	0.7	**1.1** d	**2.8**	**6.6**
Bm1_29350	ecdysone receptor	14944.m00537	4.0^d^	20.2	12.2	4.2	6.3	13.4
Bm1_36820	GTP-binding protein SAR1	BMC03183, TC2902	3.0	10.0	8.7	2.7	8.4	9.1
Bm1_44725	GTP-binding nuclear protein RAN/TC4	W05384, TC3046	2.1	4.4	3.4	2.0	4.0	6.8
Bm1_56360	Nematode cuticle collagen N-terminal domain containing	BMC12506	3.4	0.5	26.6	1.7^d^	15.8	31.5

*: Units for the Microarray experiments are in fold-changes of the treated versus untreated worms.

**: Units for the qPCR experiments are in fold changes in mRNA levels over the control group calculated from Ct values.

a The RNA used in qPCR 7d-1 is the same as the RNA from the 7 d microarray experiment.

b The RNA used in qPCR 7d-2 is a biological replicate (i.e. different from the 7 d microarray experiment and from the qPCR 7d-1).

c The RNA used in qPCR 14d-1, qPCR 14d-2 and 14 d microarray experiment is different for each (biological triplicates).

d These fold changes were not considered significant in the microarray analyses either because the *P*-value was above 0.05 or the channel rfu value (raw) was not above the cut-off of 500. These expression data were thus not included in Table S2.

Although the microarray and qRT-PCR data were in general agreement regarding the effect of tetracycline treatment, the magnitude of the changes in gene expression reported by the qRT-PCR was consistently larger than those reported by the microarray. This suggests that the qRT-PCR might be a more sensitive assay to detect changes in transcript levels than microarray hybridization, and that the microarray might be best considered as a semi-quantitative indicator suitable for defining, but not quantifying, a change in transcript level.

A list of GO terms relevant for *B. malayi* was generated and used for comparative analyses of 7 d and 14 d post-treatment. Table S5A and Table S5B provide GO terms associated with upregulated and downregulated genes, respectively. The assignment (made using GOEAST as described in the Methods) was done in a hierarchical manner, i.e. genes could be assigned more than one GO term in this analysis. Most of the significant GO associations were found to be in the upregulated genes (Table S5A).

**Figure 4:**
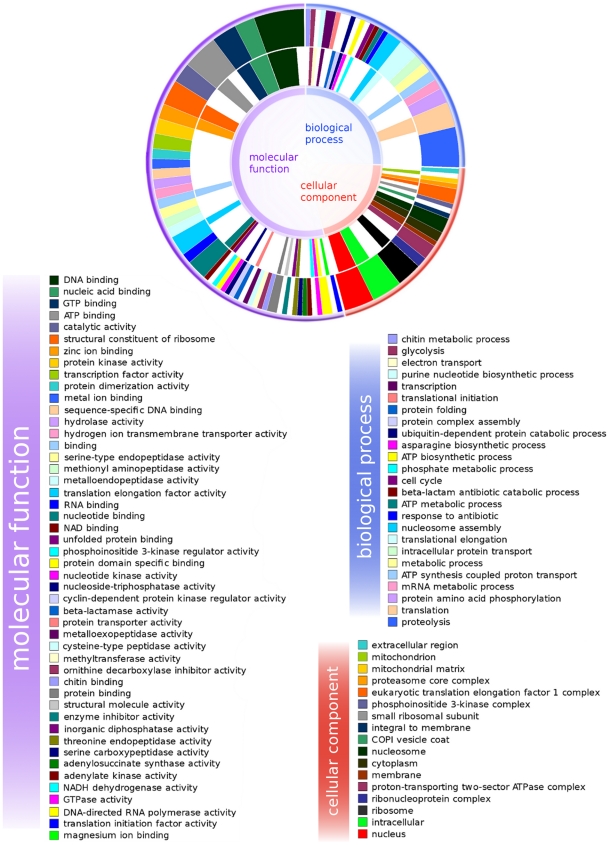
Gene Ontology used for functional classification of upregulated genes. Inner circle corresponds to 7 d and outer circle to 14 d post-treatment data. The white junction ring between the 7 d and 14 d data allows a direct comparison of the proportions between both datasets. White spaces between colored boxes indicate missing GO categories in each of the datasets, or reduced numbers of genes belonging to that category in one of the datasets as compared to the other.

Figure 4 provides a pie chart of GO terms for 7 d (inner circle) and 14 d (outer circle) post-treatment generated using InterProScan which only assigns one GO term per gene for each top level category. From this graphic representation it also appears that translation is the predominantly upregulated biological process at 7 d, while at 14 d proteolysis along with translation was upregulated. A few GO terms corresponding to molecular function are found only at 14 d, such as catalytic activity, endopeptidase, protein kinase, transcription factor, hydrolase, and proteins with dimerization activities. On the other hand, cellular components were well conserved across both time points.

Although gene annotation and pathway analyses allow a clearer understanding of which functional pathways are affected during *Wolbachia* death, a significant proportion of the regulated genes have no known function. For example, of the genes or ESTs represented by oligos on the array, at 7 d 36% of the upregulated genes and 39% of downregulated genes code for uncharacterized proteins with no domain matches and are unique to *B. malayi*; at 14 d, 16.5% of the upregulated and 41% of the downregulated genes are uncharacterized (Tables S2A and S2B). These represent a pool of genes that will eventually need to be studied as they may be important in the endosymbiotic relationship.

### Worms cultured *in vitro* are more sensitive to drug treatment

**Figure 5:**
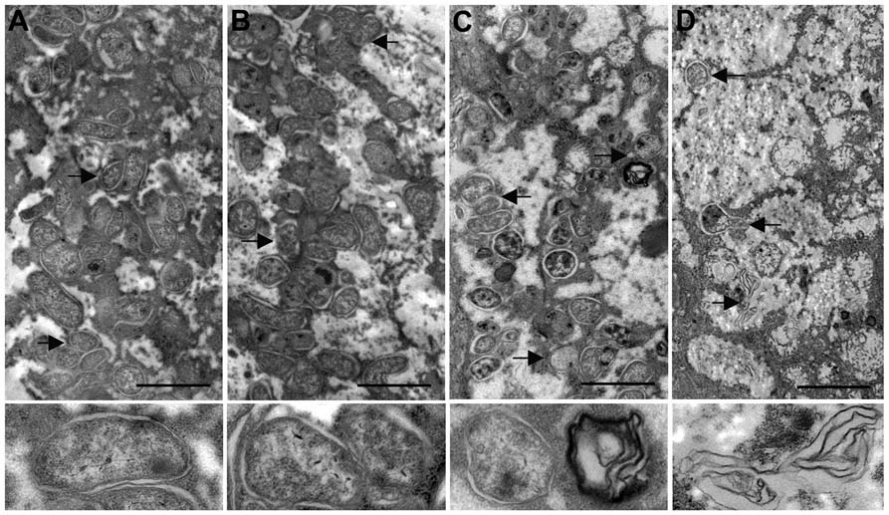
Ultrastructural observation of w*Bm* within the hypodermis of normal and tetracycline treated *B. malayi* adult female worms cultured *in vitro*. In comparison to normal worms (A) the *Wolbachia* within the hypodermal cord of worms treated for one day appeared to be normal (B), while in day 3 (C) and day 5 (D) the number of *Wolbachia* is reduced and the endobacteria appear to be degenerated; by day 5 all the endobacteria were degraded. Note that the vacuoles surrounding dead bacteria contain membrane whorls (see enlarged images C and D vs. those in A and B). Arrows point to *Wolbachia*. The bar is 2000 nm.

**Figure 6 pntd-0000525-g006:**
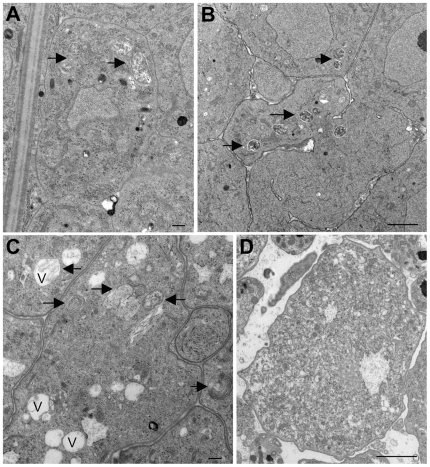
Ultrastructural observation of w*Bm* within oocytes of normal and tetracycline treated *B. malayi* adult female worms cultured *in vitro*. In comparison to untreated normal worms (A) the *Wolbachia* within oocytes of the day 1 treated worms appeared normal (B). In day 3 (C), the majority of *Wolbachia* are already degenerated and the vacuoles (v) contain little of the bacterial remnants or membrane whorls. In day 5 treated worms (D), no bacteria were found and the oocytes appeared to be completely degenerated. Arrows point to *Wolbachia*. The bar in A and C is 500 nm and the bar in B and D is 2000 nm.

**Figure 7 pntd-0000525-g007:**
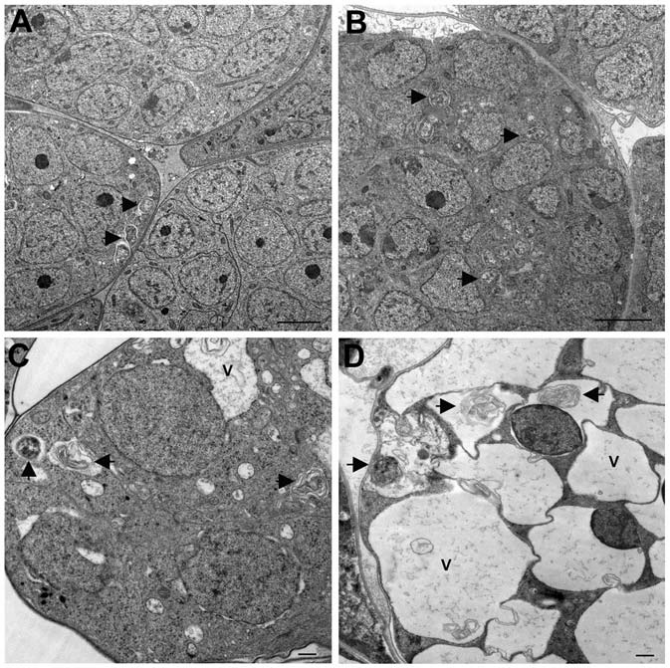
Ultrastructural observation of w*Bm* within embryos of normal and tetracycline treated *B. malayi* adult female worms cultured *in vitro*. In comparison to untreated normal worms (A) the *Wolbachia* within the embryos of the day 1 treated worms appeared normal (B). In day 3 (C), the majority of *Wolbachia* are degenerated and the vacuoles (v) contain little of the bacterial remnants or membrane whorls. In day 5 treated worms (D), enlarged vacuoles (v) are observed and they contain little of the bacterial remnants or membrane whorls. The embryos appeared also to be degenerated (D). Arrows point to *Wolbachia*. The bar in A and B is 500 nm and the bar in C and D is 2000 nm.

**Figure 8:**
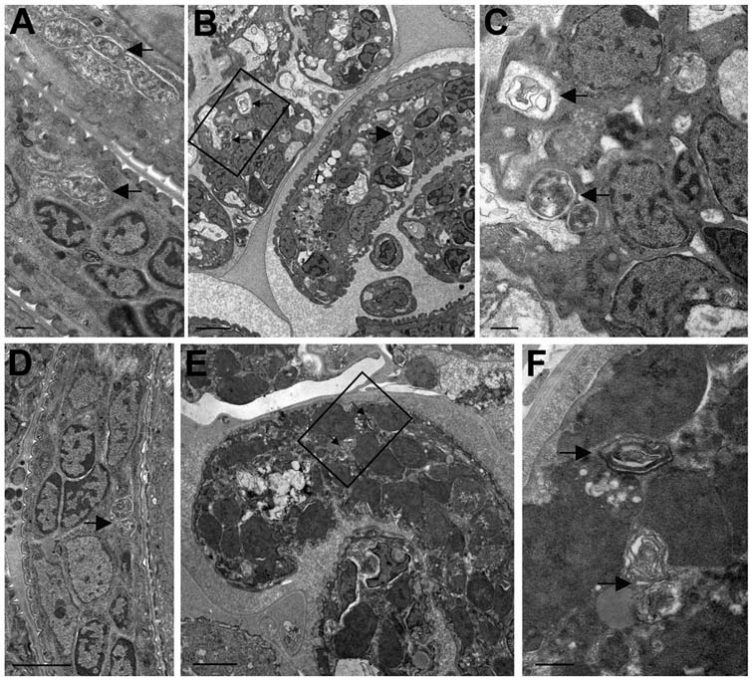
Ultrastructural observation of w*Bm* within the uterine microfilariae of normal and tetracycline treated *B. malayi* adult female worms cultured *in vitro*. In comparison to untreated normal worms (A) the *Wolbachia* within the uterine microfilariae of the day 1 treated worms appeared normal (D). In day 3 (B), the majority of *Wolbachia* are degenerated and the vacuoles contain little of the bacterial remnants or membrane whorls (C, enlarged framed area of B). In day 5 treated worms (E), the bacteria are dead and the vacuoles contain little of the bacterial remnants or membrane whorls (F, enlarged framed area of E). The microfilariae appeared also to be degenerated (E). Arrows point to *Wolbachia*. The bar in A, C and F is 500 nm and the bar in B, D and E is 2000 nm.

While the data presented above indicate that tetracycline consistently affected the *Wolbachia* endosymbiont when given to infected animals for a week, individual variability in drug uptake and availability among individual animals made it difficult to study the detailed kinetics of changes in gene expression at a time scale that was shorter than one week. To overcome the problem posed by animal to animal variation, *B. malayi* adult females were exposed to tetracycline in culture, where the process of drug exposure could be more carefully controlled. To accomplish this, female worms collected from ∼120 day infected jirds (obtained from the FR3) were cultured for 6 days in the presence or absence of 40 µg/ml tetracycline, as described in the Materials and Methods. This dose of drug was chosen as previous studies had demonstrated that this was the minimum concentration capable of reducing microfilariae release by close to 100% [18]. Initially, worms cultured for varying times in the presence or absence of tetracycline were collected and fixed for ultrastructural analysis (Figures 5–8). In untreated worms, as before, numerous *Wolbachia* were found in the hypodermal cord (Figure 5A) and in all stages of embryonic development within the uterus (Figures 6A, 7A, 8A). *Wolbachia* within the hypodermal cord of the worms treated for one day appeared to be normal (Figure 5B), while some of the bacteria had clearly degenerated by day 3 (Figure 5C) showing vacuoles with membrane whorls. By day 5 (Figure 5D) most of the bacteria were completely degenerated and morphologically resembled dead bacteria [29],[30]. The bacteria within oocytes (Figure 6B), embryos (Figure 7B) and microfilaria (Figure 8B) looked similarly normal after one day of treatment with tetracycline. However, by day three, in all three stages of development, the *Wolbachia* were completely degenerated and the majority of the vacuoles contained few bacterial remnants or membrane whorls (Figures 6C, 7C, and 8C). By day 5, not only the *Wolbachia* were completely degenerated, but the structure of the *B. malayi* oocytes (Figure 6D), embryos (Figure 7D) and microfilaria (Figure 8D) also appeared to be abnormal, degenerated and vacuolated.

### Changes in transcript levels in female *B. malayi* cultured in the presence or absence of tetracycline

**Figure 9:**
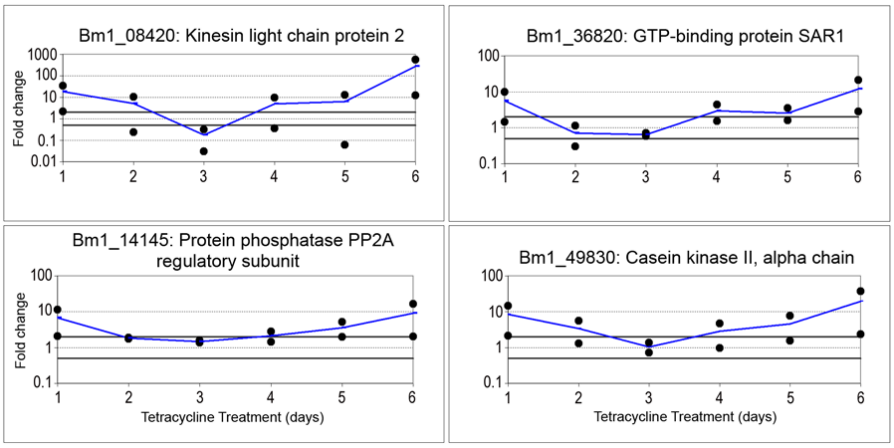
Changes in mRNA levels of selected genes from signal transduction pathways over six days of tetracycline treatment. Female *B. malayi* worms were treated with 40 µg/ml of tetracycline for up to six days. For each day, mRNA levels of treated worms were compared to the untreated control group cultured for the same period of time; the fold changes are presented in Log scale.

RNA was prepared from groups of 4 female worms each cultured for 1 to 6 days in the presence or absence of tetracycline, and used for a time course analysis of transcript levels for selected genes, using the qRT-PCR approach. Four genes that are part of the signal transduction or the signaling molecules and interaction pathways, and which were found to be regulated in the microarray and qRT-PCR experiments, were included in these studies. All four transcripts examined produced a similar pattern of gene regulation (Figure 9) and appeared to be upregulated at 1 day post *in vitro* treatment. After three days in culture, the transcript levels in the treated parasites were equivalent to or slightly less than those in the similarly cultured untreated parasites. However, after six days in culture, the transcripts in the treated parasites were again upregulated relative to those in the untreated parasites, thus exhibiting a bimodal pattern of regulation.

### Conclusion

Filarial parasites afflict hundreds of millions of individuals worldwide, and represent significant public health problems in many of the poorest countries in the world. There is a need to develop new chemotherapeutic approaches that can practically exploit the vulnerability of the human filarial parasites to the loss of the *Wolbachia*. The effects of depleting *Wolbachia* by antibiotic treatment suggest that the worms have become dependent on the bacteria for a diverse range of biological processes. From our analysis of the genomic data [43], we suspect that *B. malayi* is incapable of *de novo* purine synthesis as it lacks 9 of the 10 enzymes required to make inosine monophosphate (IMP) from phosphoribosyl pyrophosphate (PRPP). However the parasite does have the capability of converting IMP to adenosine monophosphate (AMP). Although *B. malayi* worms may be able to salvage purines from the host environment using a multitude of nucleobase and nucleoside transporters, w*Bm* appear to be able to compensate for *B. malayi*'s defective pathway by providing purines [44]. Conversely, a major role of the worm in this symbiotic relationship is likely to be provision of amino acids required for bacterial growth, since w*Bm* can only synthesize one amino acid (meso-diaminopimelate) [44]. The upregulation of translation and amino acid biosynthesis genes observed upon the depletion of *Wolbachia* by antibiotic treatment may represent some kind of feedback loop in this process. Interestingly, KEGG maps of *D. melanogaster* and its endosymbiont *Wolbachia* (Wmel) indicate that they may compensate for each other's metabolic gaps in a similar fashion [45].

*B. malayi* also lacks 6 of the 7 genes required for heme biosynthesis [43]. Heme produced by w*Bm* could be vital for worm embryogenesis and development as there is evidence that molting and reproduction are controlled by ecdysteroid-like hormones whose synthesis requires heme [46]–[48]. A recent study demonstrates that the heme biosynthetic genes in w*Bm* might be essential for *B. malayi* survival [49]. Heme is also an essential cofactor for proteins such as hemoglobins and cytochromes, among others. The depletion of heme in *B. malayi* as a result of endosymbiont cell death may in part explain the differential regulation of the cytochromes observed in our experiments.

Previous studies have identified two genes upregulated in response to anti-*Wolbachia* antibiotic treatment. Using differential display PCR, the *L. sigmodontis* phosphate permease (*Ls-ppe-1*) was identified as a tetracycline upregulated gene by days 3–6 of treatment *in vivo* and expression of this gene remained elevated through to 70 days post-treatment [50]. In another study using immunogold electron microcopy, immunohistochemistry, and qRT-PCR, it was shown that the mitochondrial heat shock protein 60 (HSP60) is upregulated in *O. volvulus* after the depletion of *Wolbachia* [51]. The increased expression of these genes in *O. volvulus* and *L. sigmondontis* in the absence of *Wolbachia* is thought to be due to a disruption of the homeostasis of the endosymbiosis. However, neither of these two genes was found to be differentially regulated in our *B. malayi* microarray experiments.

Transcriptomics is a powerful method for the identification of potential new helminth drug targets [52]. Our studies demonstrate the strength of using microarray analyses combined with bioinformatics in highlighting the pathways that are most affected by disruption of the normal endosymbiotic relationship. These pathways might not have been previously identified based on the genetic information of both the host and the endosymbiont, and thus would not be expected to be vulnerable targets of *Wolbachia* elimination. The microarray data might therefore be useful in identifying new pathways targeted by efforts to disrupt the host-endosymbiont relationship. Although the data derived from the *B. malayi* microarray were generally comparable to those derived from qRT-PCR, they appeared to be more useful in detecting general trends and pathways affected rather than providing a precise quantification of changes in the expression levels of specific genes.

Using qRT-PCR on worms that were treated *in vitro*, a bimodal pattern of gene expression was observed for the four genes tested. A similar pattern was also observed in treated *L. sigmodontis* worms [49] where it was hypothesized that the first peak was due to the effect of tetracycline on the pre-embryonic and embryonic stages of the worm (which are hypothesized to be more sensitive to the death of the endosymbiont), while the second peak was a response to the death of the endosymbiont in the hypodermal tissues of the adult. The electron microscopic studies have supported this assumption as it was observed that the oocytes and the embryos were more vulnerable to the elimination of the *Wolbachia* by tetracycline treatment. Furthermore, preliminary studies on male worms that were treated with tetracycline from 1 to 6 days indicate that for the transcripts tested there is one peak of overexpression, and it is only found at day 6 (data not shown). Since male worms lack developing embryos, this finding provides further support to the hypothesis that the bimodal pattern is related to the differential effect of tetracycline on embryos and somatic tissues in the adult female.

In summary, our studies have highlighted a few pathways and proteins that are potentially involved in the relationship between the endosymbiont w*Bm* and its *B. malayi* host. However, testing for their relevance in the symbiotic relationship will demand further characterization of these proteins and identifying putative *Wolbachia* factors that regulate their expression.

## Supporting Information

Table S1 Primer sequences for qRT-PCR experiments. List of genes and primer sequences tested by qRT-PCR.
(0.03 MB XLS)

Table S2 Upregulated and downregulated genes after 7 or 14 days of tetracycline. Table S2A lists normalized expression values and genes up or downregulated at 7 days post-treatment; Table S2B lists normalized expression values and genes up or downregulated at 14 days post-treatment.
(0.12 MB XLS)

Table S3 Specific gene transcripts regulated at 7 d and 14 d post-treatment. This table supports Figure 2. It lists normalized and log ratio expression values of genes that are regulated at both 7 and 14 days post-treatment.
(0.07 MB XLS)

Table S4 Summary of KEGG analysis. List of KEGG pathways that are targeted at 7 days or 14 days post-treatment.
(0.05 MB XLS)

Table S5 GO annotation of regulated transcripts at 7 and 14 days post-treatment. Table S5A lists GO terms associated with upregulation; Table S5B lists GO terms associated with downregulation.
(0.18 MB XLS)
